# Four Spacetime Dimensional Simulation of Rheological Waves in Solids and the Merits of Thermodynamics

**DOI:** 10.3390/e22121376

**Published:** 2020-12-05

**Authors:** Áron Pozsár, Mátyás Szücs, Róbert Kovács, Tamás Fülöp

**Affiliations:** 1 Department of Energy Engineering, Faculty of Mechanical Engineering, BME, 1521 Budapest, Hungary; paron05@gmail.com (Á.P.); szucsmatyas@energia.bme.hu (M.S.); fulop@energia.bme.hu (T.F.); 2 Montavid Thermodynamic Research Group, 1112 Budapest, Hungary; 3 Wigner Research Centre for Physics, Department of Theoretical Physics, Institute for Particle and Nuclear Physics, 1525 Budapest, Hungary

**Keywords:** symplectic numerical methods, rheology, solids, waves, spacetime, thermodynamics

## Abstract

The recent results attained from a thermodynamically conceived numerical scheme applied on wave propagation in viscoelastic/rheological solids are generalized here, both in the sense that the scheme is extended to four spacetime dimensions and in the aspect of the virtues of a thermodynamical approach. Regarding the scheme, the arrangement of which quantity is represented where in discretized spacetime, including the question of appropriately realizing the boundary conditions, is nontrivial. In parallel, placing the problem in the thermodynamical framework proves to be beneficial in regards to monitoring and controlling numerical artefacts—instability, dissipation error, and dispersion error. This, in addition to the observed preciseness, speed, and resource-friendliness, makes the thermodynamically extended symplectic approach that is presented here advantageous above commercial finite element software solutions.

## 1. Introduction

Solids may be less “solid” than expected. Beyond elastic behaviour, they may exhibit damped and delayed response. This viscoelastic/rheological reaction may not be simply explained by a viscosity-related additional stress (the Kelvin–Voigt model of rheology), but the time derivative of stress may also be needed in the description, with the simplest such model being the so-called standard or Poynting–Thomson–Zener (PTZ) one [see its details below]. Namely, the PTZ model is the simplest model that enables describing both creep (declining increase of strain during constant stress) and relaxation (declining decrease of stress during constant strain), as well as the simplest one, via which it is possible to interpret that the dynamic elasticity coefficients of rocks are different from, and larger than, their static counterpart [1,2,3,4]. Related to the latter aspect, high-frequency waves have a larger propagation speed in PTZ media than low-frequency ones [4], which makes this model relevant for, e.g., seismic phenomena and acoustic rock mechanical measurement methods.

Analytical solutions for problems in PTZ and more complex rheological solid media exist (see, e.g., [5,6]), but mostly in the force-equilibrial/quasistatic approximation, which cannot give account of transients and waves. Incorporating such ‘fast’ effects is expected to only be realizable by means of numerical calculations in most practical situations.

In many of the practical applications, such a numerical calculation must be performed many times with different material coefficients, for example, as part of a fitting procedure where the experimental data are to be fitted. Hence, the numerical scheme should be fast, resource-friendly, yet reliable and precise enough.

In addition, numerical calculations face three frequent challenges: instability (exponential blow-up of the solution), dissipation error (artificial decrease of amplitudes and energies), and dispersion error (artificial oscillations near fast changes). A good scheme keeps these artefacts under control.

Being driven by (primarily rock mechanical) applications in scope, we have tried to use commercial finite element softwares for wave phenomena in PTZ models. What we found—already for the Hookean case (but also for non-Fourier heat conduction [7])—was disappointing: the solutions ran very slowly, with large memory and CPU demand, and they were burdened by considerable numerical artefacts of the mentioned kinds.

Now, if a numerical scheme exhibits dissipation error for conservative systems, then it is expected to behave similarly for nonconservative ones, so that one cannot separate the real dissipation of mechanical energy from the dissipation artefact of the scheme. Additionally, similarly, a real wavy behaviour cannot be distinguished from the dispersion/wavy artefact.

These have motivated us to develop our own numerical scheme, which performs better [8]. Similarly to that the PTZ model can be obtained in a thermodynamical approach as an internal variable extension of Hooke elasticity [9], our starting point was a symplectic scheme for Hooke elasticity. Symplectic numerical schemes (see, e.g., [10]) provide much better large-time approximations, thanks to the fact that a symplectic numerical integrator of a conservative system is actually the exact integrator of a ‘nearby’ (coinciding in the zeroth order of the time step) conservative system.

In recent years, numerous works have been born in order to develop extensions of symplectic schemes to nonconservative systems [11,12,13,14,15,16,17,18,19,20]. We also took this path, and devised such an extension, on the example of the PTZ model, in one space dimension [8], with the novelties that some of the discretized field values reside with half space and time steps with respect to some other field values. This made the symplectic Euler method—an originally order-one accurate scheme—accurate to a second order, and the spatial accuracy was also second order.

**Figure 1:**
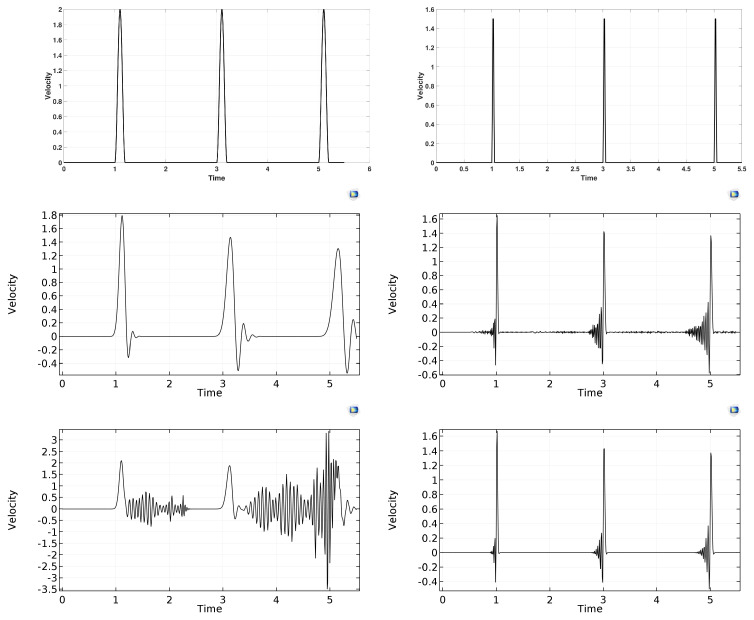
An excitation pulse, generated at the left endpoint of a finite-size one space dimensional Hookean sample, regularly arrives at the right endpoint. First row: the results from the scheme that was introduced in [8] for two different pulse lengths. Second and third rows: the corresponding results obtained by the finite element software COMSOL [8], at various settings: left column second row: Backward Differentiation Formula (BDF) Maximum order 2, third row: BDF Maximum order 5; right column second row: Dormand–Prince (DP) 5, third row: Runge–Kutta (RK) 34. In each finite element solution, dissipation error (decrease of the amplitude) and dispersion error (artificial oscillations) are both observable, even during the first three bounces. Meanwhile, the pulses in the first row keep their shape even after many bounces [8].

Indeed, our scheme has performed well: produced, in a much faster and resource-friendlier way, much more artefact-free solutions, as demonstrated in Figure 1. We note that, although the PTZ model allows for an exact integrator in the nonconservative part of the model, along analogous lines, as in [12], we have refrained from using it, since, in the future, we also wish to use the same scheme for more general nonconservative systems, so we intended to test robustness in the dissipative aspect.

In addition to comparison to finite-element solutions, in [8], we analytically derived the criteria for stability, and showed how dissipation and dispersion error can be kept small.

The first study done while using our scheme was numerically measuring wave propagation speed in one space dimensional samples, and finding good agreement with the corresponding analytical result [4].

The next step is reported here, with two novelties. The first is the generalization of the scheme to three space dimensions (3D), and the other is exploiting the whole thermodynamical theory around the PTZ model for diagnostics regarding the credibility of the numerical solution, since stability, dissipation, and dispersion error are much harder to investigate in the case of a 3D model, with its numerous vectorial and tensorial degrees of freedom.

The 3D scheme is designed in order to keep the nice, second-order, behaviour of the discretization in both the spatial and in temporal direction. Achieving this is not so trivial—different components of vectors and tensors are placed at different discretized positions in order to fulfil the aim. In parallel, the boundaries also pose a challenge: quantities must be placed in such a way that the set of equations becomes closed. We succeed in finding a rule for this that is general enough to hold for both stress and displacment boundary conditions, where these two may differ at different sides of the 3D sample.

In finding the arrangement of discretized quantities suggested here, the spacetime perspective has helped us a lot. Specifically, on one side, thermodynamical balances in their differential form are four-divergences from the spacetime aspect, and they have an integral counterpart, which, via Gauss’ theorem, helps one to find where to represent which flux-type quantity. Additionally, knowing that, from the spacetime point of view, velocity is a timelike four-vector, [21,22] gives the information that velocity should be shifted not only spatially, but also temporally. Oppositely, stress is a spacelike tensor, so no temporal shift is needed.

In parallel, thermodynamics is not only important from the aspects of balances. Namely, commercial finite element softwares only focus on the set of equations to solve, i.e., on the minimally necessary equations to follow the minimally necessary quantities. However, knowing, from the spacetime perspective, that momentum and energy form a four-quantity (also in continuum theory on Galilean spacetime) [22], in addition to the customarily taken balance of momentum, the balance of energy is also present. This enables one to follow, in addition to the mechanically considered quantities, internal energy—or, if practice favours so, temperature. The point in doing so (even in situations where thermomechanical coupling and heat conduction are neglected) is that, if, say, temperature is followed via a separate discretized evolution equation, then the conservation of total energy—at the discretized level—is not built-in, but rather is a property that will hold only approximately. Subsequently, checking how well this conservation holds along the numerical solution can provide a diagnostic tool. Thus, one may check the degree of dissipation error (i.e., the degree of violation of total energy conservation) and of dispersion error (spurious oscillations on total energy that should be a constant). This idea is demonstrated below, on the example of the PTZ model (also equipped with the thermodynamical constituents).

Furthermore, thermodynamics also provides entropy, which is known to serve as a Lyapunov function, ensuring asymptotic stability (see, e.g., [23]). Now, stability also becomes challenged at the numerical level. Accordingly, entropy, and the related entropy production, may serve as an aid for reliable numerical calculations. Certain efforts in this direction have already been made [11,12]. Here, we introduce another way of utilizing this general idea.

Specifically, we focus on entropy production. At the continuum level, it must be positive definite according to the second law of thermodynamics. However, when discretized, this property may also become challenged. Naturally, if an explicitly positive definite expression is discretized, then it remains positive definite. However, alternative forms—which only turn out to be positive definite when the further thermodynamical equations also hold—are not *ab ovo* positive definite and, correspondingly, may fail in being/remaining so along a numerical solution. Such forms are provided in a natural way, for instance, when the balance of entropy is connected to the balance of internal energy, such as when rheological models, like the PTZ one, are derived from the internal variable approach [9]. Here, we discretize such an expression of entropy production and show that its value becoming negative can forecast a loss of stability and blowing-up of the solution.

## 2. The Continuum PTZ Model and the Thermodynamics Behind

We consider a homogeneous and isotropic solid, in the small-deformation approximation (with respect to an inertial reference system), due to which we do not have to differentiate between Eulerian and Lagrangian position or make a distinction between spatial spacetime vectors, covectors, tensors, etc, as well as material manifold related ones, mass density ϱ can also be treated as constant, and the relationship between the symmetric strain tensor ε to the velocity field **v** is

(1) $$\begin{array}{c} \frac{\partial \varepsilon }{\partial t}=\frac{1}{2}\left(\overset{\to }{\nabla }⊗v+v⊗\overset{\leftarrow }{\nabla }\right), \end{array}$$

with $\overset{\to }{\nabla }$ and $\overset{\leftarrow }{\nabla }$ denoting the spatial derivative operation acting to the right and left, respectively.

The stress tensor σ is also assumed to be symmetric, and it governs the time evolution of **v** according to

(2) $$\begin{array}{c} \varrho \frac{\partial v}{\partial t}=\sigma \cdot \overset{\leftarrow }{\nabla } \end{array}$$

With the deviatoric and spherical parts of tensors,

(3) $$\begin{array}{c} \sigma^{\mathrm{sph}}=\frac{1}{3}\left(\mathrm{tr}\sigma \right)1,\;\sigma^{\mathrm{dev}}=\sigma -\sigma^{\mathrm{sph}},\;\;\varepsilon^{\mathrm{sph}}=\frac{1}{3}\left(\mathrm{tr}\varepsilon \right)1,\;\varepsilon^{\mathrm{dev}}=\varepsilon -\varepsilon^{\mathrm{sph}} \end{array}$$

(1 denoting the unit tensor), the Hooke elasticity can be expressed as

(4) $$\begin{array}{c} \sigma^{\mathrm{dev}}=E^{\mathrm{dev}}\varepsilon^{\mathrm{dev}},\;\sigma^{\mathrm{sph}}=E^{\mathrm{sph}}\varepsilon^{\mathrm{sph}},\;\;E^{\mathrm{dev}}=2G,\;E^{\mathrm{sph}}=3K, \end{array}$$

and its PTZ generalization (among its vast literature, see [4,9,24] for its treatment in the irreversible thermodynamical internal variable approach and [25], for its presentation in the GENERIC (General Equation for the Non- Equilibrium Reversible–Irreversible Coupling) framework), is

(5) $$\begin{array}{c} \sigma^{\mathrm{dev}}+\tau^{\mathrm{dev}}\frac{\partial \sigma^{\mathrm{dev}}}{\partial t}=E^{\mathrm{dev}}\varepsilon^{\mathrm{dev}}+{\hat{E}}^{\mathrm{dev}}\frac{\partial \varepsilon^{\mathrm{dev}}}{\partial t},\;\sigma^{\mathrm{sph}}+\tau^{\mathrm{sph}}\frac{\partial \sigma^{\mathrm{sph}}}{\partial t}=E^{\mathrm{sph}}\varepsilon^{\mathrm{sph}}+{\hat{E}}^{\mathrm{sph}}\frac{\partial \varepsilon^{\mathrm{sph}}}{\partial t}, \end{array}$$

the coefficients will be treated as constants hereafter.

In order to make the subsequent formulae more intelligible, we introduce

(6) $$\begin{array}{c} {\hat{\sigma }}^{\mathrm{dev}}=\sigma^{\mathrm{dev}}-E^{\mathrm{dev}}\varepsilon^{\mathrm{dev}},\;{\hat{\sigma }}^{\mathrm{sph}}=\sigma^{\mathrm{sph}}-E^{\mathrm{sph}}\varepsilon^{\mathrm{sph}} \end{array}$$

and the coefficient combinations

(7) $$\begin{array}{c} {\hat{I}}^{\mathrm{dev}}={\hat{E}}^{\mathrm{dev}}-\tau^{\mathrm{dev}}E^{\mathrm{dev}},\;{\hat{I}}^{\mathrm{sph}}={\hat{E}}^{\mathrm{sph}}-\tau^{\mathrm{sph}}E^{\mathrm{sph}}, \end{array}$$

with the aid of which (5) gets simplified to

(8) $$\begin{array}{c} {\hat{\sigma }}^{\mathrm{dev}}+\tau^{\mathrm{dev}}\frac{\partial {\hat{\sigma }}^{\mathrm{dev}}}{\partial t}={\hat{I}}^{\mathrm{dev}}\frac{\partial \varepsilon^{\mathrm{dev}}}{\partial t},\;{\hat{\sigma }}^{\mathrm{sph}}+\tau^{\mathrm{sph}}\frac{\partial {\hat{\sigma }}^{\mathrm{sph}}}{\partial t}={\hat{I}}^{\mathrm{sph}}\frac{\partial \varepsilon^{\mathrm{sph}}}{\partial t}. \end{array}$$

Taking (also for simplicity) a constant ‘isobaric’ specific heat $c_{\sigma }$ as well as neglected thermal expansion and heat conduction, the internal variable approach puts the following thermodynamical background behind the PTZ model: after eliminating the internal variable, its specific total energy

(9) etotal=ekinetic+ethermal+eelastic+erheological,ekinetic=12v2,eelastic=Edev2ϱtrεdev2+Esph2ϱtrεsph2,ethermal=cσT,erheological=τdev2ϱI^devtrσ^devσdev2+τsph2ϱI^sphtrσ^sphσsph2

with absolute temperature *T*, accompanied with specific entropy *s* and entropy production rate density $\pi_{s}$

(10) $$\begin{array}{cc} s & =c_{\sigma }\ln\frac{T}{T_{\mathrm{ref}}}, \end{array}$$

(11)πs=1T1I^devtrσ^devE^dev∂ε∂tdev−τdev∂σ∂tdev+1I^sphtrσ^sphE^sph∂ε∂tsph−τsph∂σ∂tdev(12)=1T1I^devtrσ^devσdev2+1I^sphtrσ^sphσsph2,

for which the specific internal energy part $e_{\mathrm{total}}-e_{\mathrm{kinetic}}$ fulfils the balance

(13) $$\begin{array}{c} \varrho \frac{\partial \left(e_{\mathrm{total}}-e_{\mathrm{kinetic}}\right)}{\partial t}=\mathrm{tr}\left(\sigma \frac{\partial \varepsilon }{\partial t}\right) \end{array}$$

and specific entropy the balance

(14) $$\begin{array}{c} \varrho \frac{\partial s}{\partial t}=\pi_{s}, \end{array}$$

as can be found along the lines of [9] (including its Appendix B), and it is straightforward to check. Because of the second law of thermodynamics,

(15) $$\begin{array}{c} {\hat{I}}^{\mathrm{dev}}>0,\;{\hat{I}}^{\mathrm{sph}}>0 \end{array}$$

and

(16) $$\begin{array}{c} \pi_{s}\geq 0 \end{array}$$

follow for the PTZ model [9], where (16) is already apparent from the form (12). (Recall that heat conduction is neglected, so there are no heat and entropy flux terms in the balances. In parallel, there is no term in $e_{\mathrm{total}}$ that couples *T* and ε, and—correspondingly—there is no ε dependent term in *s*, due to neglected thermal expansion.)

Superficially, it seems redundant to also provide $\pi_{s}$ in the equivalent form (11). However, it is just this not-automatically-positive-definite form that will prove to be beneficial in the diagnostics of the numerical solution.

From either balance (13) or (14), the time derivative of temperature can also be expressed:

(17) $$\begin{array}{c} \frac{\partial T}{\partial t}=\frac{T}{\varrho c_{\sigma }}\pi_{s}. \end{array}$$

As a simple analysis of the PTZ model, for ‘slow’ processes, which is to be understood with respect to the time scales

(18) $$\begin{array}{c} \tau^{\mathrm{dev}},\;{\hat{\tau }}^{\mathrm{dev}}={\hat{E}}^{\mathrm{dev}}/E^{\mathrm{dev}},\;\tau^{\mathrm{sph}},\;{\hat{\tau }}^{\mathrm{sph}}={\hat{E}}^{\mathrm{sph}}/E^{\mathrm{sph}}, \end{array}$$

a rule-of-thumb approximation is to neglect the time derivative terms (to only keep the lowest time derivative term for each quantity) in (5). The result is nothing but the Hooke model (4), for which the longitudinal and transversal wave propagation speeds are

(19) $$\begin{array}{c} c_{\mathrm{longitudinal}}=\sqrt{\frac{2E^{\mathrm{dev}}+E^{\mathrm{sph}}}{3\varrho }},\;c_{\mathrm{transversal}}=\sqrt{\frac{E^{\mathrm{dev}}}{2\varrho }}. \end{array}$$

Now, as opposed to this ‘static’ limit, let us consider the limit of ‘fast’ processes: then, it is the time derivative terms (the highest time derivative term for each quantity) that we keep. The result is the time derivative of an effective/‘dynamic’ Hooke model:

(20) $$\begin{array}{c} \sigma^{\mathrm{dev}}=E_{\infty }^{\mathrm{dev}}\varepsilon^{\mathrm{dev}},\;\sigma^{\mathrm{sph}}=E_{\infty }^{\mathrm{sph}}\varepsilon^{\mathrm{sph}},\;\;{E_{\infty }}^{\mathrm{dev}}={\hat{E}}^{\mathrm{dev}}/\tau^{\mathrm{dev}}>E^{\mathrm{dev}},\;{E_{\infty }}^{\mathrm{sph}}={\hat{E}}^{\mathrm{sph}}/\tau^{\mathrm{sph}}>E^{\mathrm{sph}}, \end{array}$$

where the inequalities follow from (15). Accordingly, the wave propagation speeds

(21) $$\begin{array}{c} {\hat{c}}_{\mathrm{longitudinal}}=\sqrt{\frac{2{\hat{E}}_{\infty }^{\mathrm{dev}}+{\hat{E}}_{\infty }^{\mathrm{sph}}}{3\varrho }}>c_{\mathrm{longitudinal}},\;{\hat{c}}_{\mathrm{transversal}}=\sqrt{\frac{{\hat{E}}_{\infty }^{\mathrm{dev}}}{2\varrho }}>c_{\mathrm{transversal}} \end{array}$$

follow. This, on one side, illustrates how the PTZ model can interpret that the dynamic elasticity coefficients of rocks are larger than their static counterpart [1,2,3,4]. On the other side, the nontrivial—frequency dependent, therefore, dispersive—wave propagation indicates that the numerical solution of PTZ wave propagation problems should contain the minimal possible amount of dispersion error, in order to give account of the dispersive property of the continuum model itself. In parallel, the dissipative nature of the PTZ model requires the minimal possible amount of dissipation error to reliably describe the decrease of wave amplitudes.

## 3. The Numerical Scheme

We take a Cartesian grid with spacings Δx, Δy, Δz, and time step Δt. Corresponding to the continuum formula (2), we introduce the finite difference discretization

**Figure 2:**
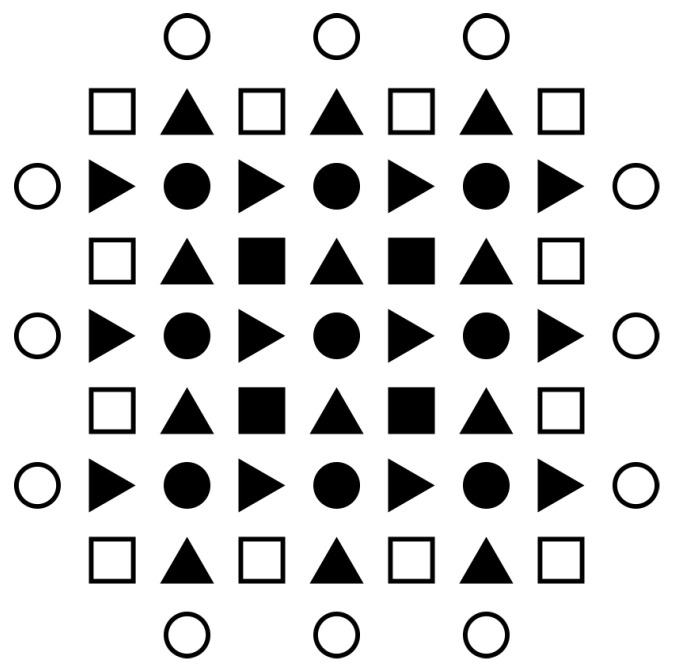
Spatial arrangement of the discretized quantities (two-dimensional projection). The circles stand for diagonal tensor components, squares for offdiagonal ones, and triangles for vector components, different components with differently oriented triangles. Void quantities are prescribed by boundary condition (in the case stress boundary conditions are considered, like here.)

(22) ϱvxl+12,m,nj+12−vxl+12,m,nj−12Δt=σxxl+1,m,nj−σxxl,m,njΔx+σxyl+12,m+12,nj−σxyl+12,m−12,njΔy+σxzl+12,m,n+12j−σxzl+12,m,n−12jΔz,

(23) ϱvyl,m+12,nj+12−vyl,m+12,nj−12Δt=σyxl+12,m+12,nj−σyxl−12,m+12,njΔx+σyyl,m+1,nj−σyyl,m,njΔy+σyzl,m+12,n+12j−σyzl,m+12,n−12jΔz,

(24) ϱvzl,m,n+12j+12−vzl,m,n+12j−12Δt=σzxl+12,m,n+12j−σzxl−12,m,n+12jΔx+σzyl,m+12,n+12j−σzyl,m−12,n+12jΔy+σzzl,m,n+1j−σzzl,m,njΔz,

where the time index *j* refers to a value at $t^{j}=j\cdot \Delta t$, j+12 to a value at tj+12=j+12·Δt, the space index *l* refers to a value at $x_{l}=l\cdot \Delta x$, *m* is the space index in the *y* direction, and *n* in the *z* direction. Accordingly, stress (and strain) values reside at integer time instants, while the velocity ones are shifted in time by half; diagonal stress (and strain) components reside at integer positions, off-diagonal ones are shifted in the two directions that match with the two Cartesian indices; and, velocity components are only shifted in the direction matching with their Cartesian index (see Figure 2). From these formulae, the j+12 indexed velocities can be expressed explicitly (as functions of earlier quantities).

This same pattern—distribution of quantities—is used for the discretization of (2):

(25) εxxl,m,nj+1−εxxl,m,njΔt=vxl+12,m,nj+12−vxl−12,m,nj+12Δx,

(26) εyyl,m,nj+1−εyyl,m,njΔt=vyl,m+12,nj+12−vyl,m−12,nj+12Δx,

(27) εzzl,m,nj+1−εzzl,m,njΔt=vzl,m,n+12j+12−vzl,m,n−12j+12Δx,

(28) εxyl+12,m+12,nj+1−εxyl+12,m+12,njΔt=12vxl+12,m+1,nj+12−vxl+12,m,nj+12Δy+vyl+1,m+12,nj+12−vyl,m+12,nj+12Δx,

(29) εxzl+12,m,n+12j+1−εxzl+12,m,n+12jΔt=12vxl+12,m,n+1j+12−vxl+12,m,nj+12Δz+vzl+1,m,n+12j+12−vzl,m,n+12j+12Δx,

(30) εyzl,m+12,n+12j+1−εyzl,m+12,n+12jΔt=12vyl,m+12,n+1j+12−vyl,m+12,nj+12Δz+vzl,m+1,n+12j+12−vzl,m,n+12j+12Δy,

from which formulae the j+1 indexed strains can be explicitly expressed, and for the discretized version of (5),

(31) $$\begin{array}{cc} \alpha {\left(\sigma_{pq}^{\mathrm{dev}}\right)}_{l^{\prime },m^{\prime },n^{\prime }}^{j} & +\left(1-\alpha \right){\left(\sigma_{pq}^{\mathrm{dev}}\right)}_{l^{\prime },m^{\prime },n^{\prime }}^{j+1}+\frac{{\left(\sigma_{pq}^{\mathrm{dev}}\right)}_{l^{\prime },m^{\prime },n^{\prime }}^{j+1}-{\left(\sigma_{pq}^{\mathrm{dev}}\right)}_{l^{\prime },m^{\prime },n^{\prime }}^{j}}{\Delta t}\\ \; & =E^{\mathrm{dev}}\left[\alpha {\left(\varepsilon_{pq}^{\mathrm{dev}}\right)}_{l^{\prime },m^{\prime },n^{\prime }}^{j}+\left(1-\alpha \right){\left(\varepsilon_{pq}^{\mathrm{dev}}\right)}_{l^{\prime },m^{\prime },n^{\prime }}^{j+1}\right]+{\hat{E}}^{\mathrm{dev}}\frac{{\left(\varepsilon_{pq}^{\mathrm{dev}}\right)}_{l^{\prime },m^{\prime },n^{\prime }}^{j+1}-{\left(\varepsilon_{pq}^{\mathrm{dev}}\right)}_{l^{\prime },m^{\prime },n^{\prime }}^{j}}{\Delta t}, \end{array}$$

(32) $$\begin{array}{cc} \alpha {\left(\sigma_{pq}^{\mathrm{sph}}\right)}_{l^{\prime },m^{\prime },n^{\prime }}^{j} & +\left(1-\alpha \right){\left(\sigma_{pq}^{\mathrm{sph}}\right)}_{l^{\prime },m^{\prime },n^{\prime }}^{j+1}+\frac{{\left(\sigma_{pq}^{\mathrm{sph}}\right)}_{l^{\prime },m^{\prime },n^{\prime }}^{j+1}-{\left(\sigma_{pq}^{\mathrm{sph}}\right)}_{l^{\prime },m^{\prime },n^{\prime }}^{j}}{\Delta t}\\ \; & =E^{\mathrm{sph}}\left[\alpha {\left(\varepsilon_{pq}^{\mathrm{sph}}\right)}_{l^{\prime },m^{\prime },n^{\prime }}^{j}+\left(1-\alpha \right){\left(\varepsilon_{pq}^{\mathrm{sph}}\right)}_{l^{\prime },m^{\prime },n^{\prime }}^{j+1}\right]+{\hat{E}}^{\mathrm{sph}}\frac{{\left(\varepsilon_{pq}^{\mathrm{sph}}\right)}_{l^{\prime },m^{\prime },n^{\prime }}^{j+1}-{\left(\varepsilon_{pq}^{\mathrm{sph}}\right)}_{l^{\prime },m^{\prime },n^{\prime }}^{j}}{\Delta t},\\ \; & p,q=x,y,z,\;l^{\prime },m^{\prime },n^{\prime }=\mathrm{integers}\;\mathrm{or}\;\mathrm{half}-\mathrm{integers}\;\mathrm{depending}\;\mathrm{on}\;p,q, \end{array}$$

where α=1/2 ensures second-order accuracy of the whole scheme (the proof is analogous to the one in [8]), from which— together with (3)—the j+1 indexed stresses can be explicitly expressed, except for stress boundary locations, where we express strain (and know stress from the boundary condition).

Actually, regarding boundary conditions, the rule we found for both stress boundary condition and velocity (or displacement) boundary condition is that, if a quantity is missing for determining another boundary quantity, then that missing quantity is to be added outside the boundary. This also works for mixed boundary conditions, with different ones meeting at edges of a rectangular sample, for example. In what follows, we present stress boundary condition examples (relevant, e.g., for a wide class of rock mechanical applications).

The pattern of which quantity to reside where—at integer or half-integer space and time indices—could also be conjectured from the structure of the equations, but, as said in the Introduction, the spacetime viewpoint helps a lot to find this arrangement a geometrically—spacetime geometrically— natural one.

In the reversible special case of the Hooke system, this scheme is symplectic. It is actually the symplectic Euler method (in words: ‘new${}_{1}$ from old${}_{1}$ and old${}_{2}$, new${}_{2}$ from new${}_{1}$ and old${}_{2}$’). The interpretation is the improvement: here, new${}_{1}$ and new${}_{2}$ are shifted in time with respect to each other, so second-order accuracy is achieved, while the conventional interpretation of the symplectic Euler method is first-order only. In parallel, since mechanical energy (the Hamiltonian) is a velocity dependent term plus a strain dependent term (stress becomes a simple linear function of strain), our scheme is explicit. This also remains true at the PTZ level, so one can expect—and find, actually—a fast-running program code.

For the aspects of thermodynamics, we also discretize (17) [here using the form (12)], explicitly expressing the j+12 indexed temperature values from

(33) Tl,m,nj+12−Tl,m,nj−12Δt=1ϱcσ1I^devtrσ^devσdev2l,m,nj+1I^sphtrσ^sphσsph2l,m,nj,

where the notation (6) is utilized, and the traces are to be expanded in Cartesian components and the terms containing offdiagonal components—that reside at half space-shifted locations in two indices—are averaged around the location l,m,n, first neighbours only.

## 4. Solution for a Rectangular Beam, and the Role of Total Energy

**Figure 3:**
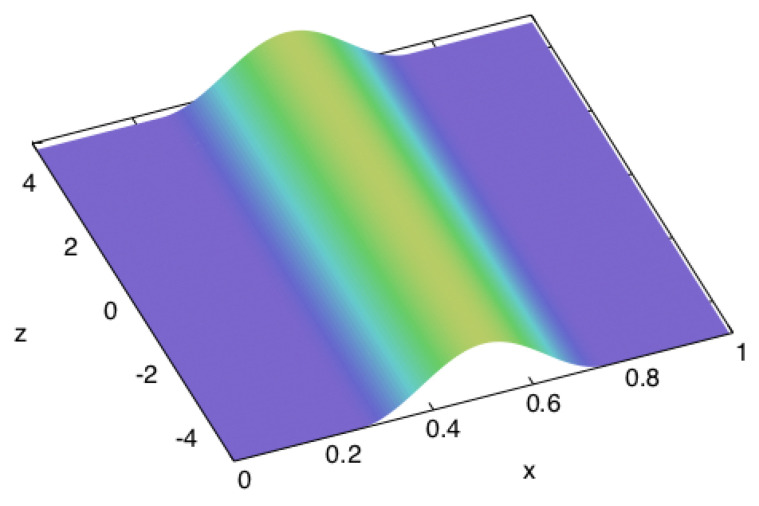
The spatial distribution of normal stress as the boundary condition on one side of a square cross-sectioned beam that is infinitely long in the *z* direction. The excitation is a single cosine-shaped ‘bump’ in time, too.

The first example on which we demonstrate the scheme is a square cross-sectioned long beam, being thus treated as a plane-strain problem. Initially, the beam is in relaxed/equilibrium state (zero stress, strain and velocity, and homogeneous temperature). Subsequently, on one of its sides, a single normal stress pulse is applied, with profile

(34) $$\begin{array}{c} \sigma_{yy}\left(t,x,0,z\right)=\left\{\begin{array}{cc} \sigma_{b}\left\{\frac{1}{2}\left[1-\cos\left(2\pi \frac{t}{\tau_{b}}\right)\right]\cdot \frac{1}{2}\left[1-\cos\left(2\pi \frac{x-X/2}{W_{b}}\right)\right]\right\}\; & \mathrm{if}\;\;0\leq t\leq \tau_{b}\;\;\mathrm{and}\\ & \frac{X-W_{b}}{2}\leq x\leq \frac{X+W_{b}}{2},\\ 0\; & \mathrm{otherwise} \end{array}\right. \end{array}$$

(see Figure 3), where $\tau_{b}$ is the duration of the pulse, $\sigma_{b}$ is its amplitude, $W_{b}$ is its spatial width, and *X* is the width of the beam. On the other sides, normal stress is constantly zero (free surfaces).

A 50×50 grid is considered in the *x*–*y* plane, for 240 time steps, where the time step is the largest at which stability is maintained. Notably, stability investigation is fairly involved for this problem and it requires a separate whole study. Further settings (in appropriate units) are ϱ=1, $E^{\mathrm{dev}}=4$, $E^{\mathrm{sph}}=10$, $\tau_{b}=0.3$, $\sigma_{b}=5$, $W_{b}/X=0.6$.

### 4.1. Hooke Case

**Figure 4:**
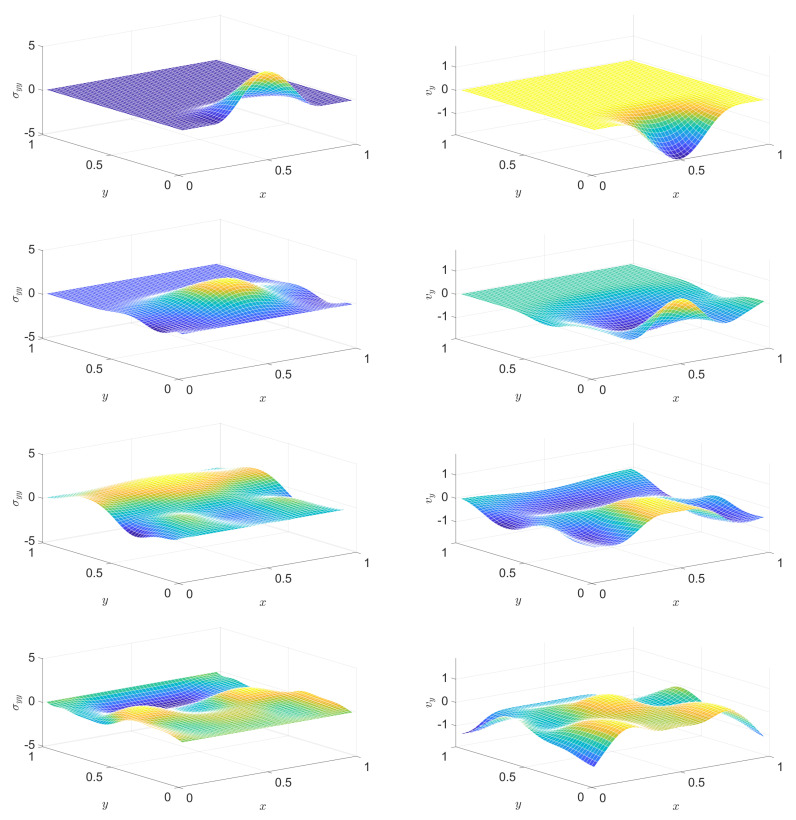
Distribution of a stress component (left column) and of a velocity component (right column) at various instants, in the Hooke case. From top to bottom: snapshots at instants $\left(1/2\right)\tau_{b}$, $\tau_{b}$, $\left(3/2\right)\tau_{b}$, $2\tau_{b}$, respectively.

**Figure 5:**
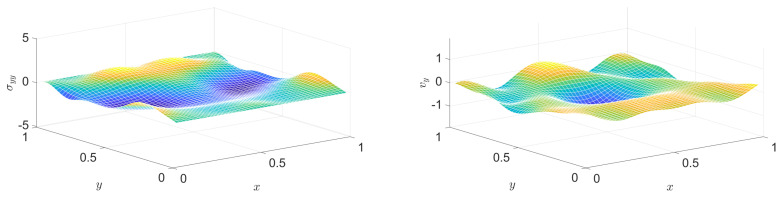

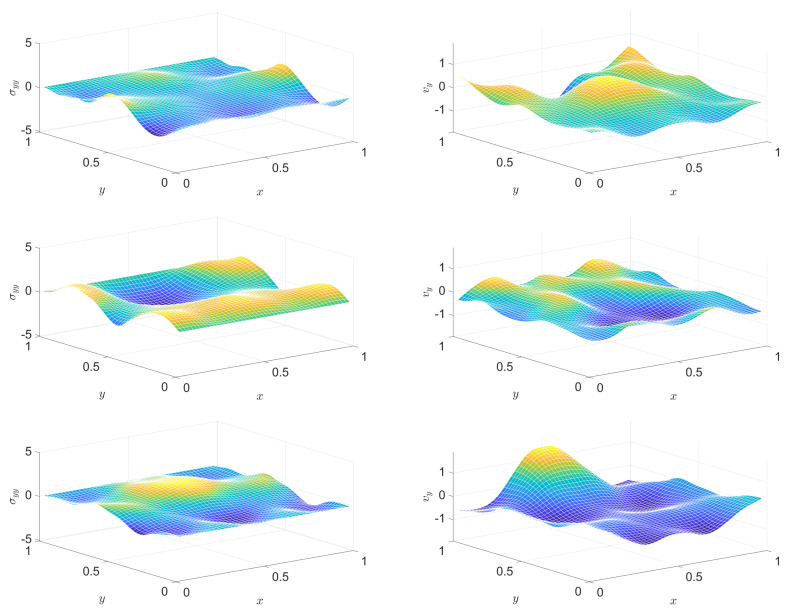
Continuation of Figure 4: distribution of a stress component (left column) and of a velocity component (right column) at various instants, in the Hooke case. From top to bottom: snapshots at instants $\left(5/2\right)\tau_{b}$, $3\tau_{b}$, $\left(7/2\right)\tau_{b}$, $4\tau_{b}$, respectively.

Figure 4 and Figure 5 show snapshots of a stress component distribution and a velocity component.

In a movie format, it is more spectacular how reliably the simulation performs.

Furthermore, it is not only the eye that could judge the reliability: with the help of thermodynamics, energy—in the Hooke case, mechanical energy— proves to be a useful diagnostic tool:

- if it explodes then there is instability;
- if it deviates from a constant then there is dissipation error; and,
- if it is wavy/oscillating then there is dispersion error.

**Figure 6:**
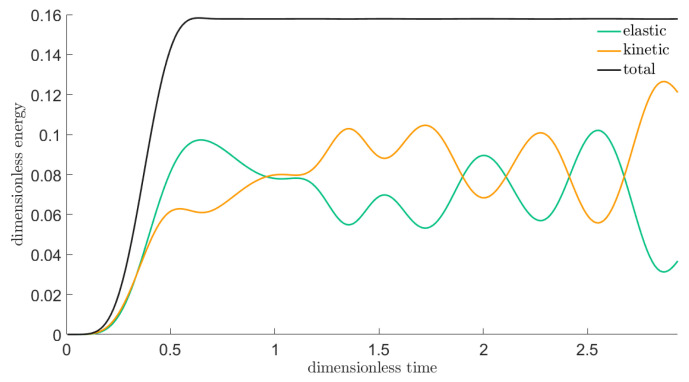
Mechanical energy types as functions of time, for the Hooke case.

The scheme presented here also functions satisfactorily in this aspect, as displayed in Figure 6. For energy, we perform summation, over the integer centred discrete cells, of the energy terms discretized along the above lines, including that averages, like for (33), are taken wherever necessary, also in the time direction (for kinetic energy).

### 4.2. PTZ Case

**Figure 7:**
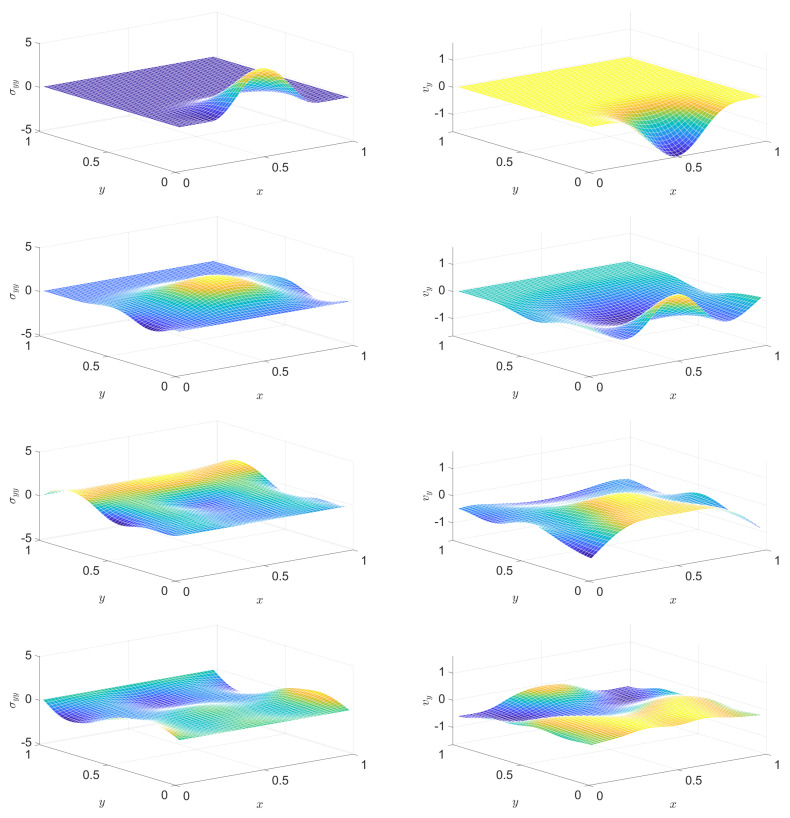
Distribution of a stress component (left column) and of a velocity component (right column) at various instants, in the Poynting–Thomson–Zener (PTZ) case. From top to bottom: snapshots at instants $\left(1/2\right)\tau_{b}$, $\tau_{b}$, $\left(3/2\right)\tau_{b}$, $2\tau_{b}$, respectively.

**Figure 8:**
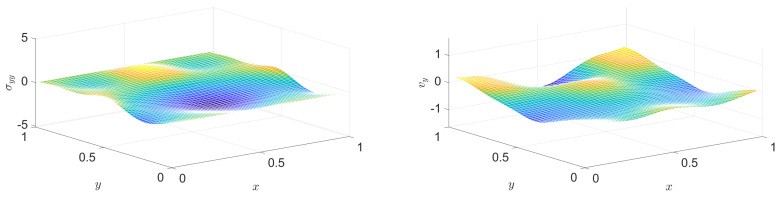

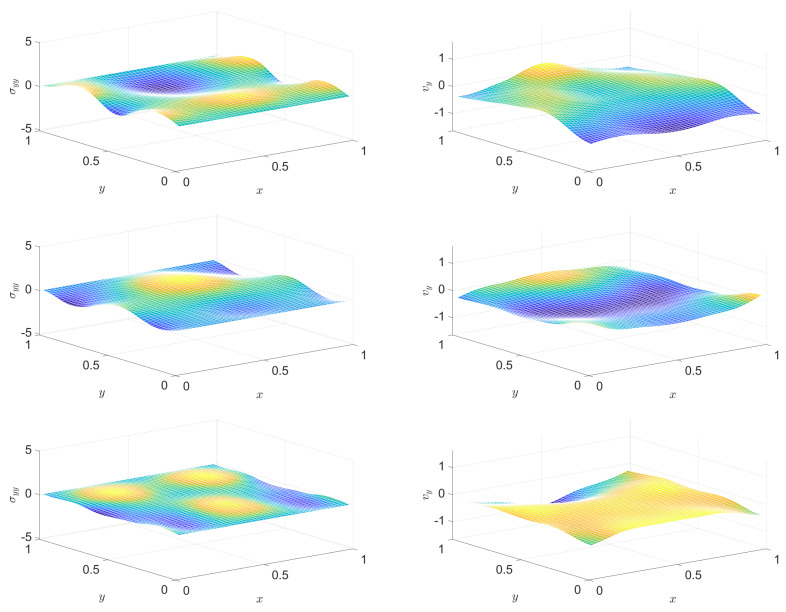
Continuation of Figure 7: distribution of a stress component (left column) and of a velocity component (right column) at various instants, in the PTZ case. From top to bottom: snapshots at instants $\left(5/2\right)\tau_{b}$, $3\tau_{b}$, $\left(7/2\right)\tau_{b}$, $4\tau_{b}$, respectively.

**Figure 9:**
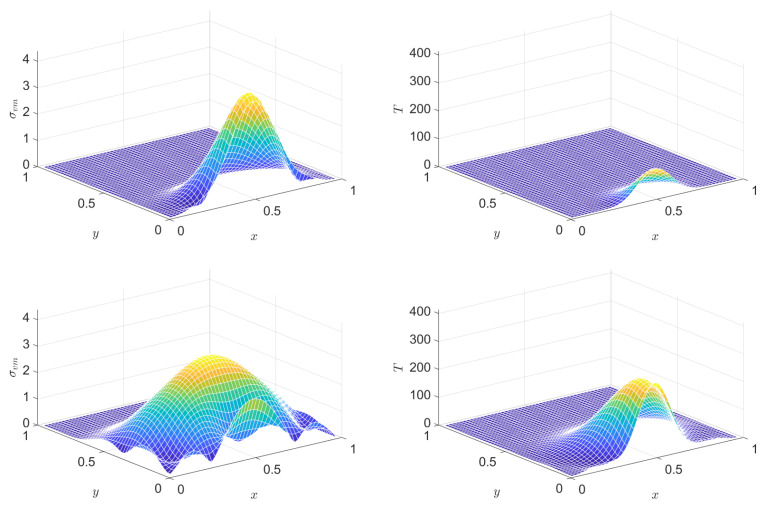

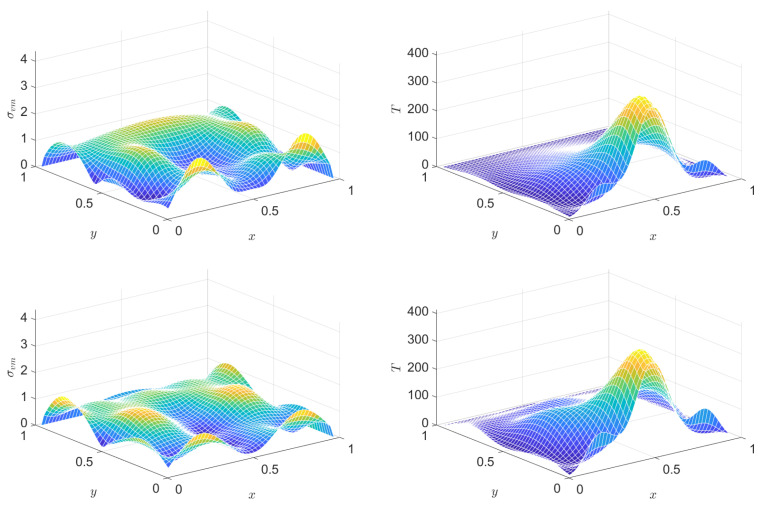
Snapshots of the distribution of the stress invariant $\sqrt{\mathrm{tr}({\hat{\sigma }}^{{\mathrm{dev}}^{2}})}$ (left column) and of temperature (right column) at various instants, in the PTZ case. From top to bottom: snapshots at instants $\left(1/2\right)\tau_{b}$, $\tau_{b}$, $\left(3/2\right)\tau_{b}$, $2\tau_{b}$, respectively.

**Figure 10:**
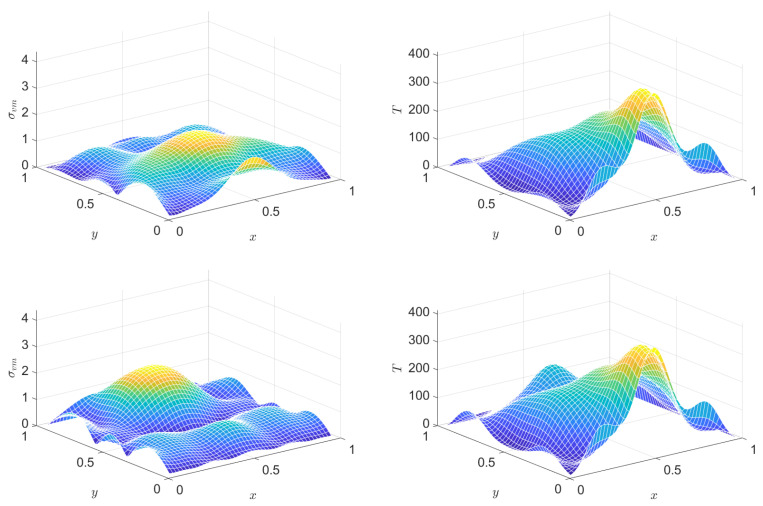

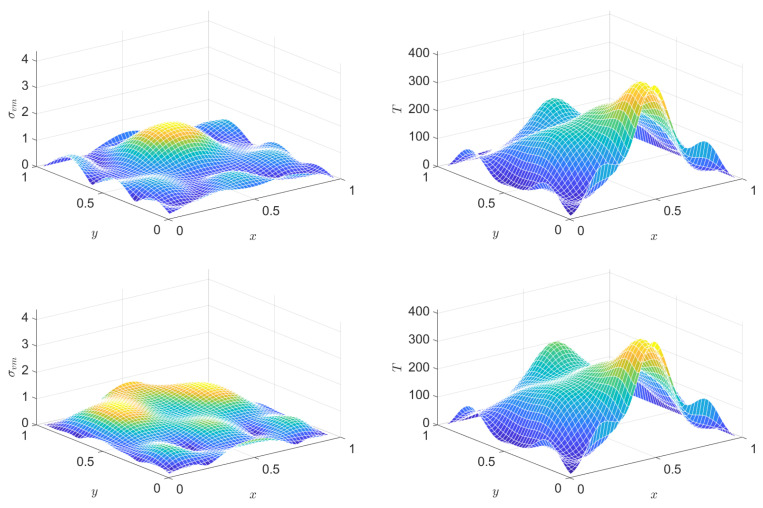
Continuation of Figure 9: Snapshots of the distribution of the stress invariant $\sqrt{\mathrm{tr}({\hat{\sigma }}^{{\mathrm{dev}}^{2}})}$ (left column) and of temperature (right column) at various instants, in the PTZ case. From top to bottom: snapshots at instants $\left(5/2\right)\tau_{b}$, $3\tau_{b}$, $\left(7/2\right)\tau_{b}$, $4\tau_{b}$, respectively.

In a PTZ medium, the solution of the analogous problem is similarly good. Figure 7, Figure 8, Figure 9 and Figure 10 present snapshots, where Figure 9 and Figure 10 display two further quantities: trσ^devσdev2 (essentially the Huber–Mises–Hencky or von Mises equivalent stress) and temperature. Dissipation is nicely indicated via temperature. Settings (in addition to the above ones for the Hooke case) are ${\hat{E}}^{\mathrm{dev}}=1.4$, $\tau^{\mathrm{dev}}=0.2$, $c_{\sigma }=0.001$, $T^{0}=0.1$, α=12.

**Figure 11:**
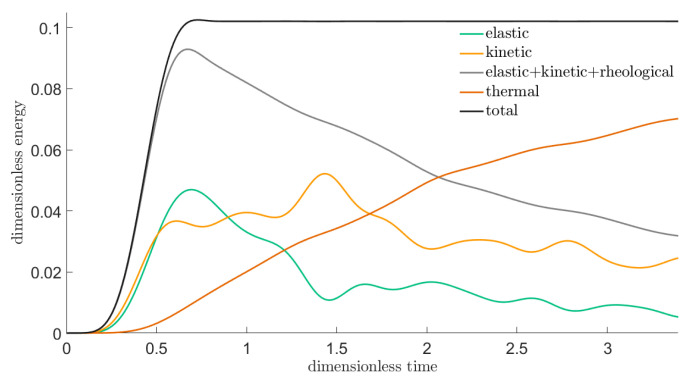
Total energy and the various energy types as functions of time, for the PTZ case.

The diagnostic role of the various energies, and especially their sum, is a great help again for checking whether the simulation performs acceptably. Figure 11 illustrates how the scheme that is introduced above behaves in this respect.

## 5. Solution for a Cube, and the Role of Entropy Production

**Figure 12:**
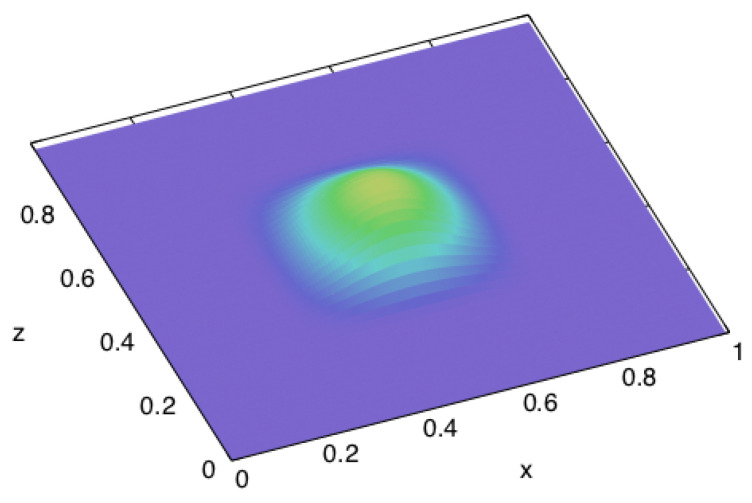
The spatial distribution of normal stress as the boundary condition on one side of a cube. The excitation is also a single cosine-shaped ‘bump’ in time.

In the second example treated, a cube is considered, initially relaxed, as in the previous example, and then one of its sides being pressed by a cosine ‘bump’ in time, as well as in both spatial directions:

(35) σyyt,x,0,z=σb{121−cos2πtτb·121−cos2πx−X/2Wbσb{121−cos2πtτb·121−cos2πz−X/2Wb}if0≤t≤τbandX−Wb2≤x,z≤X+Wb2,0otherwise

(see Figure 12), where the notations are analogous to the ones of the previous case: $\tau_{b}$ is the duration of the pulse, $\sigma_{b}$ is its amplitude, $W_{b}$ is its spatial width, and *X* is the length of the edges of the cube. On the other sides, normal stress is constantly zero (free surfaces), like in the previous example.

Here, we only present the PTZ model, on a 25×25×25 grid, with all other settings being the same as in the previous example.

**Figure 13:**
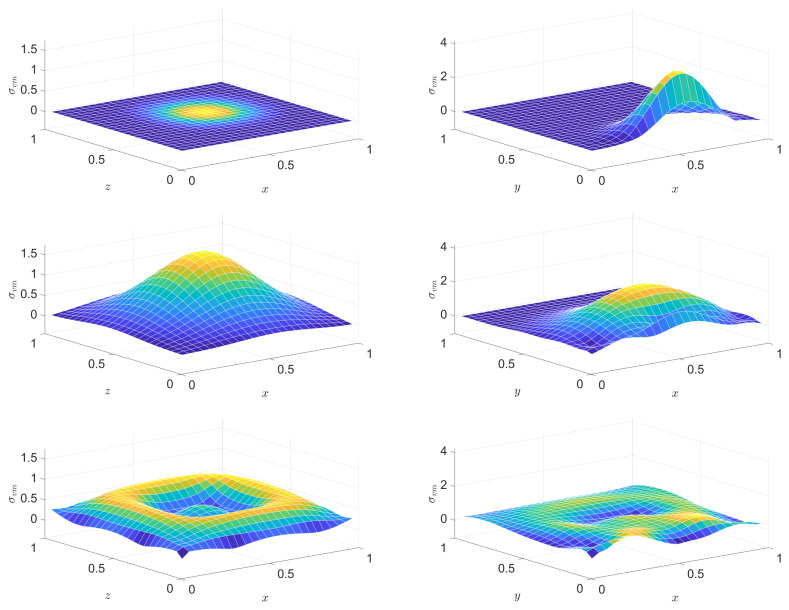

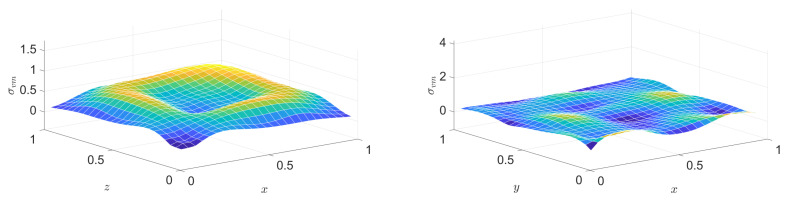
Snapshots of the distribution of the stress invariant $\sqrt{\mathrm{tr}({\hat{\sigma }}^{{\mathrm{dev}}^{2}})}$ at the mid-plane parallel to the excited side (left column) and at a mid-plane orthogonal to it (right column) at various instants, in the PTZ case. From top to bottom: snapshots at instants $\left(1/2\right)\tau_{b}$, $\tau_{b}$, $\left(3/2\right)\tau_{b}$, $2\tau_{b}$, respectively.

**Figure 14:**
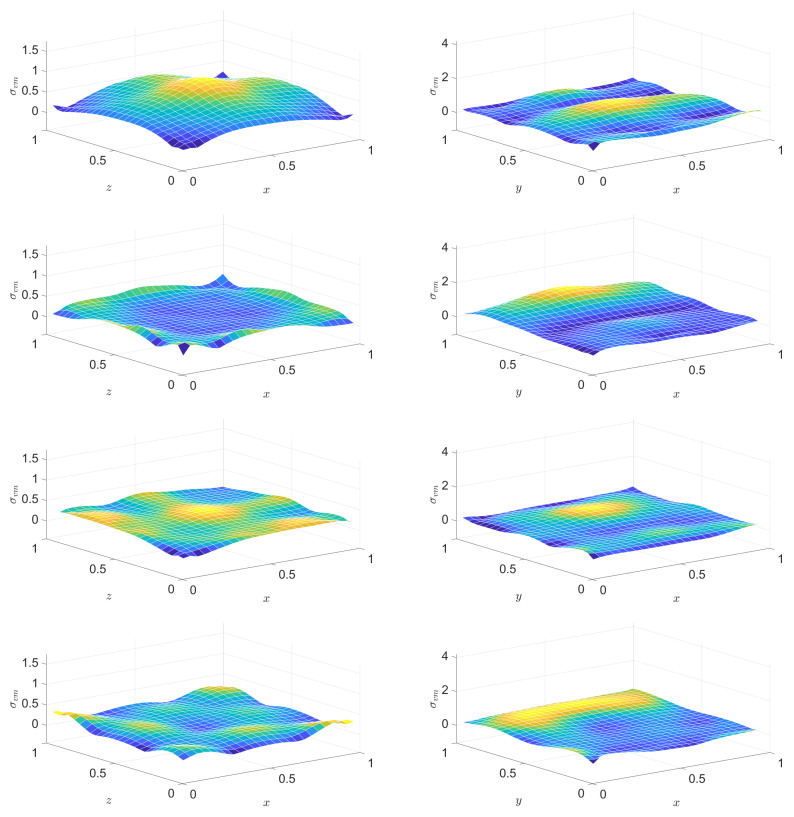
Continuation of Figure 13: snapshots of the distribution of the stress invariant $\sqrt{\mathrm{tr}({\hat{\sigma }}^{{\mathrm{dev}}^{2}})}$ at the mid-plane parallel to the excited side (left column) and at a mid-plane orthogonal to it (right column) at various instants, in the PTZ case. From top to bottom: snapshots at instants $\left(5/2\right)\tau_{b}$, $3\tau_{b}$, $\left(7/2\right)\tau_{b}$, $8\tau_{b}$, respectively.

The solution proves similarly satisfatory, as in the former case. Figure 13 and Figure 14 show snapshots of the distribution of the von Mises stress invariant on two mid-planes of the cubes.

Next, in this example, we demonstrate the usefulness of another thermodynamical quantity, entropy production rate density.

It is instructive to start with showing how the idea works in the simpler, one space dimensional, setting (the rod discussed in [8]). The one space dimensional analogue of (11) is

(36) $$\begin{array}{cc} \pi_{s} & =\frac{1}{T}\frac{1}{\hat{I}}\;\hat{\sigma }\cdot \left(\hat{E}\frac{\partial \varepsilon }{\partial t}-\tau \frac{\partial \sigma }{\partial t}\right) \end{array}$$

Let us introduce four different discretizations of this product, embodying the patterns

- old·(new−old),
- old·(new−older),
- new·(new−old), and
- new·(new−older):

(37) $$\begin{array}{cc} \; & \frac{1}{T_{n}^{j}}\frac{1}{\hat{I}}\;{\hat{\sigma }}_{n}^{j}\cdot \left(\hat{E}\frac{\varepsilon_{n}^{j+1}-\varepsilon_{n}^{j}}{\Delta t}-\tau \frac{\sigma_{n}^{j+1}-\sigma_{n}^{j}}{\Delta t}\right), \end{array}$$

(38) $$\begin{array}{cc} \; & \frac{1}{T_{n}^{j}}\frac{1}{\hat{I}}\;{\hat{\sigma }}_{n}^{j}\cdot \left(\hat{E}\frac{\varepsilon_{n}^{j+1}-\varepsilon_{n}^{j-1}}{2\Delta t}-\tau \frac{\sigma_{n}^{j+1}-\sigma_{n}^{j-1}}{2\Delta t}\right), \end{array}$$

(39) $$\begin{array}{cc} \; & \frac{1}{T_{n}^{j}}\frac{1}{\hat{I}}\;{\hat{\sigma }}_{n}^{j+1}\cdot \left(\hat{E}\frac{\varepsilon_{n}^{j+1}-\varepsilon_{n}^{j}}{\Delta t}-\tau \frac{\sigma_{n}^{j+1}-\sigma_{n}^{j}}{\Delta t}\right), \end{array}$$

(40) $$\begin{array}{cc} \; & \frac{1}{T_{n}^{j}}\frac{1}{\hat{I}}\;{\hat{\sigma }}_{n}^{j+1}\cdot \left(\hat{E}\frac{\varepsilon_{n}^{j+1}-\varepsilon_{n}^{j-1}}{2\Delta t}-\tau \frac{\sigma_{n}^{j+1}-\sigma_{n}^{j-1}}{2\Delta t}\right), \end{array}$$

where $T_{n}^{j}$ denotes the time average Tnj−12+Tnj+12/2.

**Figure 15:**
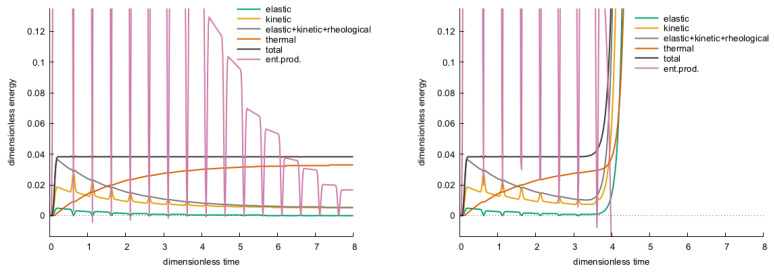

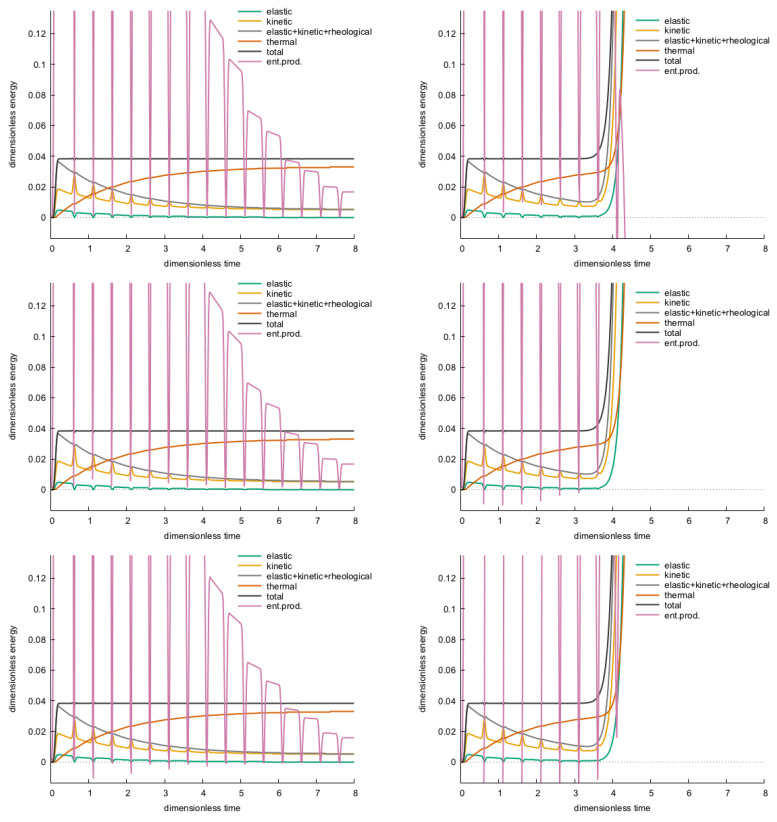
Entropy production rate according to the four discretized versions (37)–(40), respectively, for a time step resulting in a stable run (left column) (namely, when the rheological Courant number $\hat{c}\Delta t/\Delta x$ is 1, where $\hat{c}=\sqrt{\hat{E}/\left(\tau \varrho \right)}$ is the high-frequency rheological wave propagation speed) and for a choice leading to an unstable outcome (right column) (rheological Courant number $\hat{c}\Delta t/\Delta x=1.003$). PTZ model with $\tau E/\hat{E}=0.25$; the sample length *X* is such that $\hat{E}/E=5X/c$ with the low-frequency/elastic wave propagation speed $c=\sqrt{E/\varrho }$. See [8] for further details.

These four versions are integrated in space and plotted in Figure 15, the left column for a stable setting and the right column for an unstable one. The energies are also displayed. Only 25 space cells have been chosen to enhance artefacts.

Visibly, certain versions become negative when instability gets exposed. Moreover, some become negative even before that, showing, at an early stage, that there is a problem to come.

**Figure 16:**
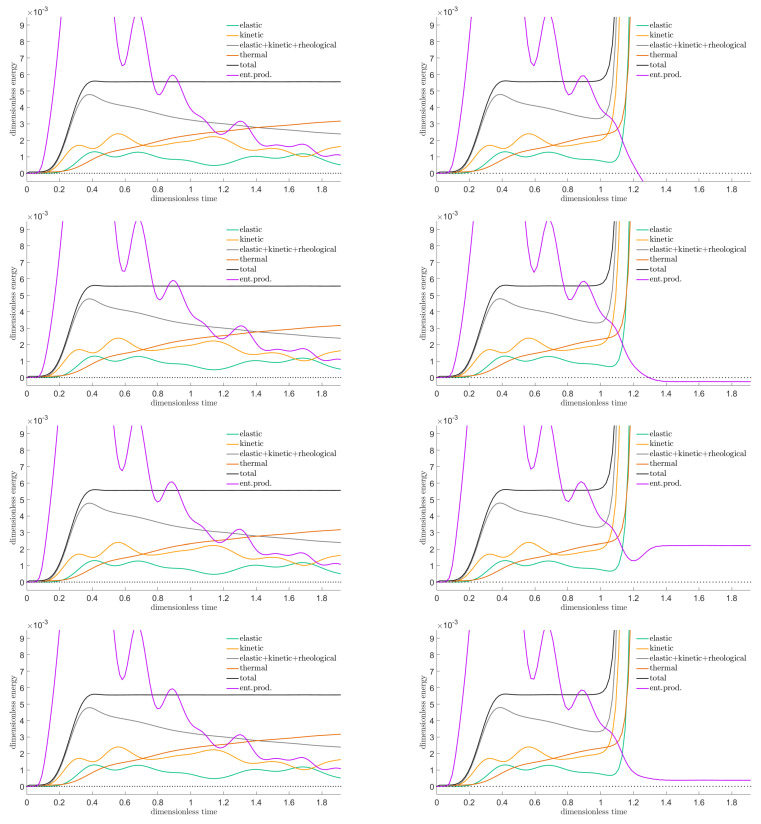
Entropy production rate according to the four discretized versions (37)–(40) generalized to three space dimensions, respectively, computed for a PTZ cube, with a time step resulting in a stable run (left column) and for a choice leading to an unstable outcome (right column).

Next, let us see how the three space dimensional generalizations behave for the problem of the pressed cube: the outcomes can be seen in Figure 16. An intentionally rude 10×10×10 grid is taken, the time unit is divided to 125 time steps in order to lead to a stable solution and to 100 time steps producing an unstable one. Further settings are ϱ=1, $E^{\mathrm{dev}}=3$, $E^{\mathrm{sph}}=5$, ${\hat{E}}^{\mathrm{dev}}=20$, $\tau^{\mathrm{dev}}=0.391$, $c_{\sigma }=0.001$, $T^{0}=0.1$, $\tau_{b}=0.25$, and $\sigma_{b}=3$, $W_{b}/X=0.6$, α=12.

One can see that an appropriately chosen discretization diagnoses instability.

Such numerical comparisons between stable and unstable parameter domains, which are probably enhanced by some analytical considerations, can help in the future in deciding which discretized entropy production rate is useful for diagnosing what.

## 6. Two-Dimensional Wave Propagation According to the Finite Element Software COMSOL

In [8], a comparison of solutions via the one space dimensional scheme with corresponding finite element solutions was presented. Repeating it for the present three space dimensional scheme would be informative. We found it advisable to start investigating the higher dimensional wave propagation describing the possibilities of finite element softwares in a much simpler setting than the one treated above, based on the (discouraging) experience that is gained with the one-dimensional case, and since a rheological model like PTZ in the deviatoric–spherical formulation is difficult to translate to the capabilities of classic finite element softwares.

Namely, we consider the wave equation in two spatial dimensions, for a single scalar degree of freedom u=u(t,x,y), with constant (unit) coefficients and no source term in the equation:

(41) $$\begin{array}{c} \frac{\partial^{2}u}{\partial t^{2}}=\frac{\partial^{2}u}{\partial x^{2}}+\frac{\partial^{2}u}{\partial y^{2}}. \end{array}$$

A rectangular sample is taken with ‘flux’ (Neumann) boundary condition (normal spatial derivative component is prescribed): on one of the edges, one cosine-type pulse of the kind (34) is applied, while the other edges are kept ‘free’ (zero normal derivative). Initially, both *u* and ∂u/∂t are set to zero (‘relaxed initial state’).

**Figure 17:**
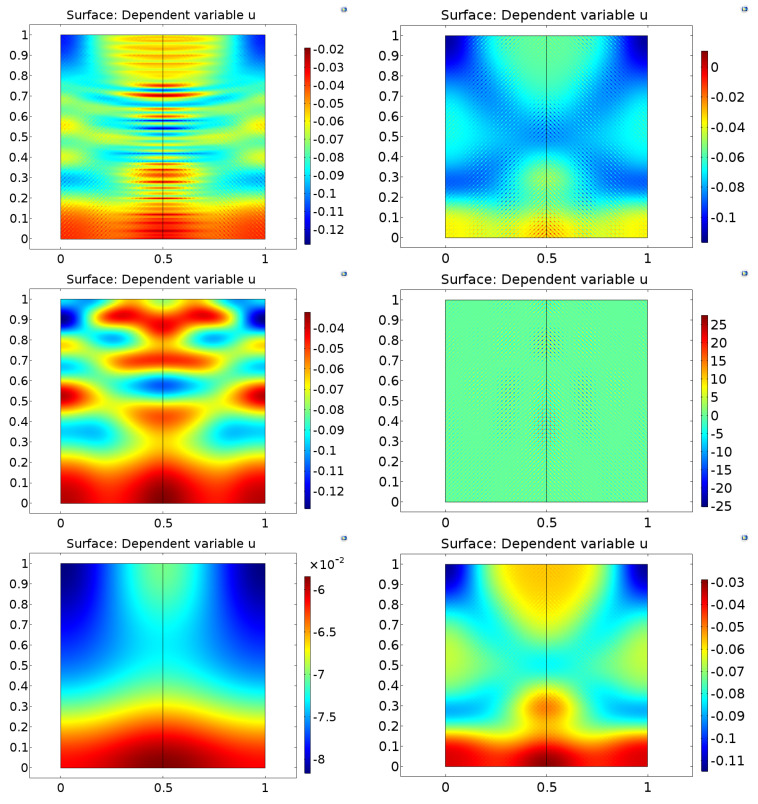

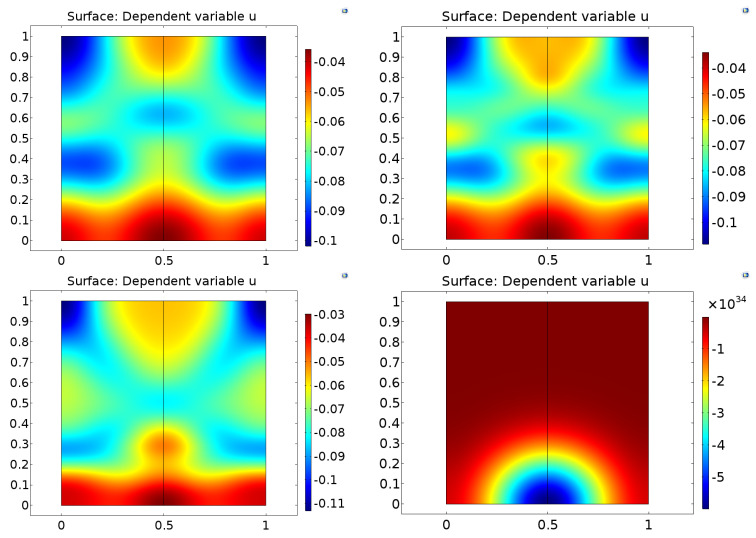
u(2,x,y) with various COMSOL settings (cf. Figure 1). Left column, from top to bottom: BDF Max. order 5 & min. order 2, BDF Max. order 3 & min. order 2, BDF Max. order 1 & min. order 1, BDF Max. order 5 & min. order 3 Strict time stepping, RK34 Default. Right column, from top to bottom: DP5 PI-Smooth, DP5 PI-Standard, DP5 PI-Quick, Generalized-α (GA), GA Predictor Constant. Not shown (failed like DP5 PI-Standard or GA Predictor Constant): DP5 Pi-Off, RK34 Manual, Cash-Karp 5 Free or Strict (Manual with a small time step gives a result similar to that of RK Default).

To stay similar to the numerical calculations that are presented here, a 50 × 50 spatial grid is taken. The software that we use is COMSOL v5.3a, where this wave equation problem is a built-in possibility. The pulse duration is 0.3 time unit. Figure 17 displays the resulting spatial distribution of the scalar degree of freedom after two units of time, obtained with various available settings of COMSOL v5.3a.

One can see that the solution depends on the settings very seriously. There are large-scale differences and fine-structured irregularities. Moreover, one has no means of validation which outcome is correct to what extent.

When taking into account that our full problem has 16 coupled degrees of freedom in three spatial dimensions and with further time derivatives (in the PTZ model), it does not seem to be reasonable to try to represent it via any commercial finite element software as long as such a much simpler problem cannot be confidently treated.

## 7. Discussion

The numerical scheme presented here, due to its symplectic root, second-order accuracy, and the equation-friendly and spacetime geometry friendly arrangement of discretized quantities, has been found to provide reliable results in a fast and resource-friendly way. Being a finite difference scheme, it is not very flexible to simulate arbitrary shaped samples, but, already, the extension of the Cartesian formulae to cylindrical and spherical geometries promises useful applications, including the various wave-based measurement methods that are used in rock mechanics (see, e.g., [26]), many of which rely on simple and easily treatable sample shapes. Fitting a rheological model on experimental data may require many runs so good finite difference schemes find their applicability.

The investigation of stability and dissipative and dispersion error is expected to be much more involved than in the corresponding one space dimensional situation, where the analysis was done in [8]. Nevertheless, it is an important task for the future, because the outcomes support efficient applications of the scheme.

It is an interesting challenge to apply the presented scheme for other dissipative situations (like [27], just to mention one example).

A systematic and general framework could be obtained, which is also beneficial for other purposes, if the spacetime background is strengthened further, by using four-quantities, four-equations on them, and formulating discretization in a fully four-geometrical way. It would, for example, help in building connection to a finite element—spacetime finite element—approach, along which way objects of general shape could also be treated. Notably, the current finite element paradigm has deficiencies and probably needs to be renewed, as indicated by results found here and earlier works [7,8].

In addition, the use the thermodynamical full description of a system for monitoring and controlling numerical artefacts during a computer simulation is a promising perspective. The steps made here: recognizing the usefulness of total energy and its various parts, and of entropy production rate density, are hoped to contribute to a future routine in numerical environments, where thermodynamics could be utilized.
